# The Pro-Inflammatory Deletion Allele of the NF-κB1 Polymorphism Is Characterized by a Depletion of Subunit p50 in Sepsis

**DOI:** 10.3390/ijms23147559

**Published:** 2022-07-08

**Authors:** Britta Marko, Paulina Heurich, Patrick Thon, Frieda Zimmer, Lars Bergmann, Hartmuth Nowak, Katharina Rump, Björn Koos, Michael Adamzik, Matthias Unterberg, Tim Rahmel

**Affiliations:** Klinik für Anästhesiologie, Intensivmedizin und Schmerztherapie, Universitätsklinikum Knappschaftskrankenhaus Bochum, In der Schornau 23-25, D-44892 Bochum, Germany; britta.marko@kk-bochum.de (B.M.); paulina.heurich@ruhr-uni-bochum.de (P.H.); patrick.thon@ruhr-uni-bochum.de (P.T.); frieda.zimmer@kk-bochum.de (F.Z.); lars.bergmann@kk-bochum.de (L.B.); hartmuth.nowak@kk-bochum.de (H.N.); katharina.k.rump@rub.de (K.R.); bjoern.koos@ruhr-uni-bochum.de (B.K.); michael.adamzik@kk-bochum.de (M.A.); matthias.unterberg@kk-bochum.de (M.U.)

**Keywords:** nuclear factor ‘kappa-light-chain-enhancer’ of activated B-cells, NF-κB, NF-κB1 promoter polymorphism, subunit p50, sepsis, mitochondrial dysfunction

## Abstract

The functionally important NF-κB1 promoter polymorphism (−94ins/delATTG) significantly shapes inflammation and impacts the outcome of sepsis. However, exploratory studies elucidating the molecular link of this genotype-dependent pattern are lacking. Accordingly, we analyzed lipopolysaccharide-stimulated peripheral blood mononuclear cells from both healthy volunteers (*n* = 20) and septic patients (*n* = 10). All individuals were genotyped for the −94ins/delATTG NF-κB1 promoter polymorphism. We found a diminished nuclear activity of the NF-κB subunit p50 in ID/DD genotypes after 48 h of lipopolysaccharide stimulation compared to II genotypes (*p* = 0.025). This was associated with higher TNF-α (*p* = 0.005) and interleukin 6 concentrations (*p* = 0.014) and an increased production of mitochondrial radical oxygen species in ID/DD genotypes (*p* = 0.001). Although ID/DD genotypes showed enhanced activation of mitochondrial biogenesis, they still had a significantly diminished cellular ATP content (*p* = 0.046) and lower mtDNA copy numbers (*p* = 0.010) compared to II genotypes. Strikingly, these findings were mirrored in peripheral blood mononuclear cells taken from septic patients. Our results emphasize the crucial aspect of considering NF-κB subunits in sepsis. We showed here that the deletion allele of the NF-κB1 (−94ins/delATTG) polymorphism was associated with the lower nuclear activity of subunit p50, which, in turn, was associated with aggravated inflammation and mitochondrial dysfunction.

## 1. Introduction

Sepsis is defined as a dysregulated immune response to an infection, which leads to metabolic disruption, multiple organ failure, and, ultimately, death [1]. However, the precise molecular interrelations of different inflammatory patterns on the pathogenesis of organ dysfunction are still elusive. Thus, despite the greatest scientific effort, causal treatment options alleviating organ dysfunction are still lacking, keeping morbidity and mortality unacceptably high in sepsis and causing millions of deaths worldwide [2,3]. One explanatory variable is that risk factors that are able to describe the wide variability of clinical course and outcome in sepsis remain poorly understood [4].

A promising candidate as an important risk factor is the transcription factor family nuclear factor ‘kappa-light-chain enhancer’ of activated B-cells (NF-κB) [5,6]. The NF-κB family comprises five subunits (namely NF-κB1, NF-κB2, RelA, RelB, and c-Rel) with pleiotropic intrinsic effects [7]. All five NF-κB family members share structural homology with a retroviral oncoprotein (v-Rel), resulting in their classification as Rel proteins [8]. Unlike the other NF-κB family members, the NF-κB1 and NF-κB2 proteins are translated as large precursors, p105 and p100, respectively, and undergo processing regulated by the ubiquitin–proteasome pathway [8]. The processed forms of p100 and p105 (p52 and p50, respectively) do not contain a transactivation domain and need to dimerize with one of the other three family members to function as ‘active’ transcription factors [9]. Thus, homo- and hetero dimers of p50 and p52 operate as transcriptional repressors, as they can bind to promoter elements without the activation of the transcriptional machinery [9]. Of note, the inflammatory immune response and cellular stress reaction in sepsis are mainly composed of the most abundant NF-κB subunits p65 and p50 [10,11]. In addition, there is mounting evidence that particularly the composition of NF-κB subunits is a central regulator of the inflammatory response and, thus, fundamentally involved in the molecular links to organ dysfunction in sepsis [8,12]. Therefore, the proper composition of the NF-κB subunits seems to have a great impact on multiple facets of the sepsis pathophysiology, such as reactive oxygen species (ROS) production or mitochondrial dysfunction [13,14,15]. Accordingly, genetic variations that alter the composition and nuclear activity of the NF-κB subunits may have a significant impact on the outcome and mortality of sepsis.

An insertion–deletion polymorphism (−94 ins/del ATTG) in the promoter of NF-κB1 (rs28362491) was recently described as an important risk factor in sepsis and acute respiratory distress syndrome [5,6,16]. The deletion allele here is associated with an aggravated inflammatory response and, subsequently, with the severity of organ failure and even mortality in patients suffering from sepsis [5]. However, the mechanistic interrelations between the altered nuclear activity of the NF-κB subunits in context with the NF-κB1 polymorphism and its impact on inflammation and organ failure are still poorly understood. Thus, a deeper understanding of associated molecular patterns, especially regarding the nuclear activity of the NF-κB subunits, seems prudent in order to elucidate the underlying interrelations in sepsis.

Accordingly, we prospectively tested the hypotheses that the NF-κB1 insertion–deletion (−94ins/delATTG) polymorphism (1) alters the nuclear activity of NF-κB1, (2) affects the inflammatory response, and (3) is associated with the mitochondrial dysfunction in sepsis.

## 2. Results

### 2.1. Lipopolysaccharide Stimulation of PBMC from Healthy Individuals

Eight out of twenty healthy volunteers (mean age: 55.8 ± 9.1 years; 8 females and 12 males) were homozygous II genotypes, 8 were heterozygous ID genotypes, and four were homozygous DD genotypes. Heterozygous and homozygous deletion allele carriers were subsequently merged into one group as a result of the low frequency of the DD genotype.

**Figure 1:**
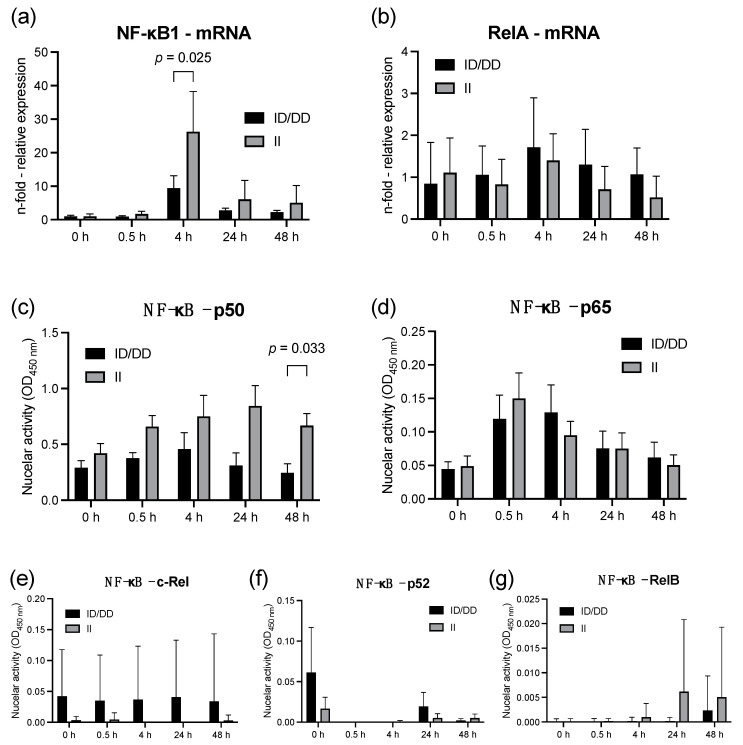
Lipopolysaccharide (LPS) decreases nuclear DNA-binding activity of NF-κB subunit p50 in deletion allele carriers compared to II genotypes. LPS stimulation of PBMCs from healthy volunteers before and after 0.5, 4, 24, and 48 h, respectively. Upper panel: mRNA expression (quantitative polymerase chain reaction) normalized to beta actin of (**a**) NF-κB1 and (**b**) RelA in LPS-stimulated PBMCs of healthy volunteers. (**c**–**g**) Nuclear binding activity of NF-κB subunits of PBMCs from healthy volunteers. Columns represent means with error bars as standard deviation. *p* values relate to post hoc Šídák’s test adjusted for multiple comparisons. No labeling of *p*-values designates no statistically significant difference at respective time points. There were no missing data.

The NF-κB1 mRNA expression reached its peak in both genotype groups after 4 h of LPS stimulation. However, D allele carriers showed only a 9.4-fold (±3.8) increase in NF-κB1 mRNA expression normalized to unstimulated controls (0 h) at this timepoint, compared with a 26.3-fold (±12.0) increase in II genotype (*p* = 0.025, Figure 1a). The diminished expression of NF-κB1 in deletion allele carriers was also reflected by the 2.8-fold lower nuclear activity of the related subunit p50 48 h after LPS stimulation (*p* = 0.033, Figure 1c). However, the mRNA expression and the nuclear activity of RelA (*p* = 0.218) and the related subunit p65 (*p* = 0.997) did not differ between II and ID/DD genotypes within 48 h after LPS stimulation (Figure 1a,d).

**Figure 2:**
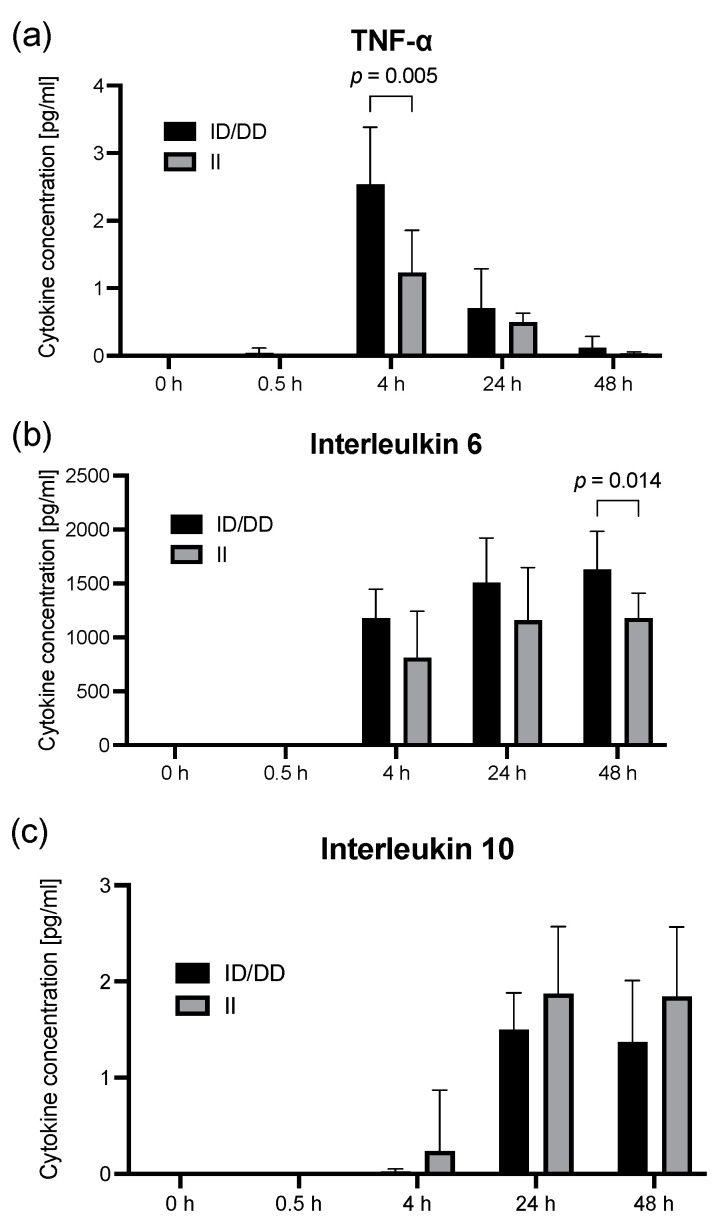
The deletion allele of the NF-κB1 promoter polymorphism is associated with an aggravated inflammation in lipopolysaccharide-stimulated PBMCs. LPS stimulation of PBMCs from healthy volunteers before and after 0.5, 4, 24, and 48 h, respectively. Concentrations of selected cytokines in PBMC cell culture supernatants. (**a**) TNF-α; (**b**) interleukin 6; (**c**) interleukin 10. Columns represent means with error bars as standard deviation. *p* values relate to post hoc Šídák’s test adjusted for multiple comparisons. No labeling of *p*-values designates no statistically significant difference at respective time points. Cytokine concentrations were derived from a calibration curve. There were no missing data.

The cytokine release in terms of an altered immune response may be affected by the activity of NF-κB1; therefore, we determined the effect of LPS on the concentration of IL-6, IL-10, and TNF-α (Figure 2). The LPS (10 μg/mL) increased the TNF-α concentration within 4 h more than 1000-fold in both groups (*p* < 0.001). Interestingly, the effect of LPS seemed to be modulated by the NF-κB 1 polymorphism because we found an approximately 2-fold larger TNF-α increase in ID/DD genotypes (2.5 ± 0.8 pg/mL) compared to II genotypes (1.2 ± 0.6 pg/mL, *p* = 0.005, Figure 2). In addition, the IL-6 concentration was also higher after 48 h of LPS stimulation in D allele carriers (1630 ± 349 pg/mL) compared to II genotypes (1180 ± 230 pg/mL, *p* = 0.014, Figure 2). By contrast, the IL-10 concentration effect of LPS was not different between the genotypes of the NF-κB 1 promoter polymorphism (*p* = 0.568).

**Figure 3:**
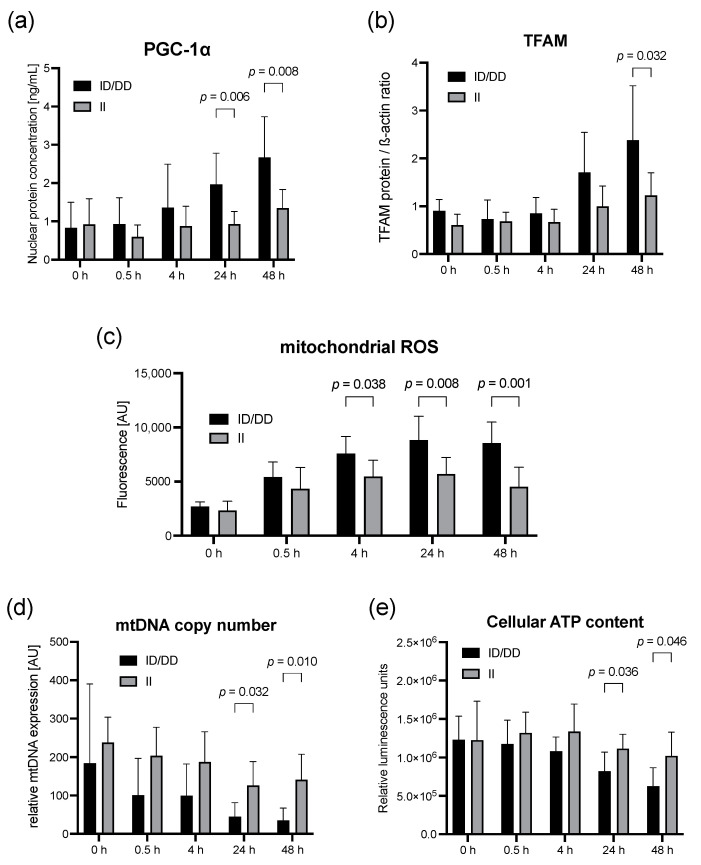
Deterioration of mitochondrial function in LPS-stimulated PBMCs from healthy volunteers is aggravated in deletion allele carriers. Upper panel: (**a**) PGC-1α (ELISA of nuclear protein extracts) in PBMCs of healthy volunteers. (**b**) TFAM protein expression in LPS-stimulated PBMCs from healthy volunteers. Middle Panel: (**c**) Mitochondrial ROS production in LPS-stimulated PBMCs from healthy volunteers. Lower panel: Time course of mitochondrial function indicators (**d**) mitochondrial DNA copy number and (**e**) cellular ATP content in PBMCs from healthy volunteers before and 0.5, 4, 24, and 48 after LPS stimulation. Cellular ATP was determined using a luciferase-based assay and expressed as relative fluorescent units normalized to 2.25 × 10^5^ cells per well. Columns represent means with error bars as standard deviation. *p* values relate to post hoc Šídák’s test adjusted for multiple comparisons. No labeling of *p*-values designates no statistically significant difference at respective time points. There were no missing data.

Exploring a genotype-dependent impact on mitochondrial dysfunction, we found a 1.4-fold higher mitochondrial ROS production in ID/DD genotypes after LPS stimulation at 4 h (*p* = 0.04), 1.6-fold higher at 24 h (*p* = 0.008), and almost 2-fold higher at 48 h (*p* = 0.001) (Figure 3c). Despite an enhanced induction of mitochondrial biogenesis (Figure 3a,b), our results still suggested an aggravated mitochondrial dysfunction in D-allele carriers. The mitochondrial DNA copy number after LPS stimulation decreased here by approximately 75% after 48 h, but only by approximately 45% in II genotypes, each compared to unstimulated controls (*p* = 0.010, Figure 3d). In addition, the cellular ATP content nearly halved within 48 h after LPS stimulation but only showed a moderate decline of 20% in II genotypes compared to unstimulated controls (*p* = 0.046, Figure 3e).

### 2.2. Findings in PBMCs Taken from Septic Patients

Baseline characteristics of the septic patients stratified by their genotype are shown in Table 1. A total of 10 septic patients were enrolled. The mean age was 58 years (±13), and 60% (6/10) of patients were male. At the time of study inclusion, 40% (4/10) were receiving mechanical ventilation, and 90% (9/10) were taking vasopressors. The mean SOFA (Sepsis-related Organ Failure Assessment) score at baseline was 10 (±3), and more than 30% (3/10) of the patients fulfilled the Sepsis-3 criteria for septic shock at the time of admission.

Upon admission to the intensive care unit, II genotypes (9 ± 6) and ID/DD genotypes (11 ± 4) showed a comparable disease severity represented by their SOFA scores (*p* = 0.668; Table 1). In addition, no differences were found between II and ID/DD genotypes regarding the baseline serum C-reactive protein (*p* = 0.305), mechanical ventilation (*p* = 0.778), vasopressor support (*p* = 0.646), or serum lactate concentration (*p* = 0.817; Table 1). Moreover, there were no statistically significant differences in other baseline characteristics between II and ID/DD NF-κB1 genotypes (Table 1).

**Figure 4:**
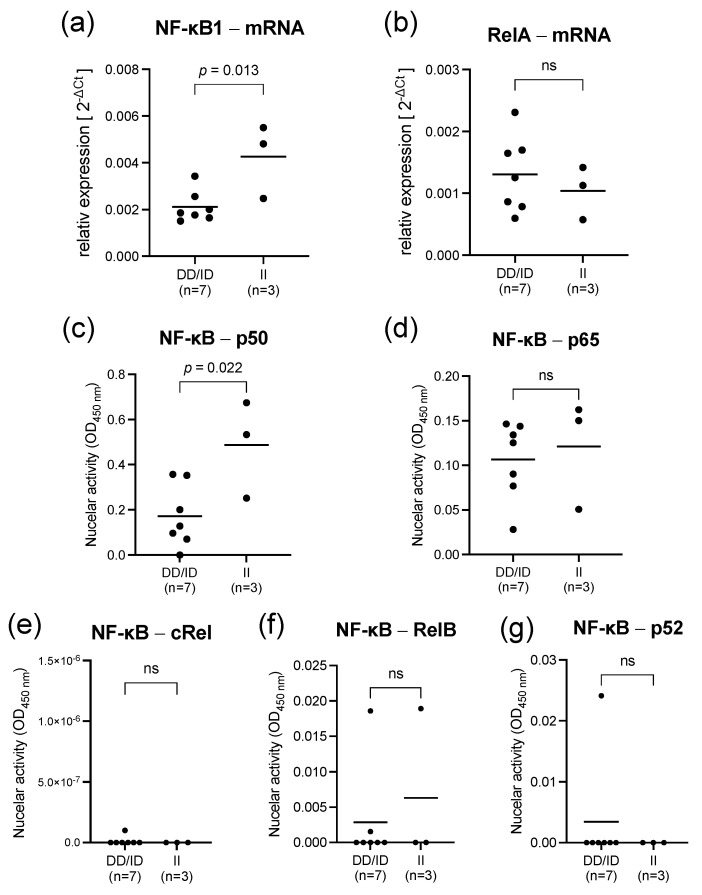
Sepsis is associated with a diminished DNA binding activity of NF-κB subunit p50 in deletion allele carriers. Results representing PBMCs from septic patients (*n* = 10) sampled within 24 h after the diagnosis and stratified according to II-genotypes and ID/DD genotypes of the NF-κB1 promoter polymorphism. Upper panel: mRNA expression (quantitative polymerase chain reaction) normalized to beta actin of (**a**) NF-κB1 and (**b**) RelA in PBMCs of septic patients. (**c**–**g**) Nuclear binding activity of NF-κB subunits of sepsis patients. Each dot represents an individual patient; line represents mean. There were no missing data. *p*-values were determined using the Mann–Whitney test; ns designates no statistically significant difference. Cytokine concentrations pg/mL were derived from a calibration curve.

At the time of admission to the intensive care unit, D allele carriers showed a 2-fold lower NF-κB1 mRNA expression compared to II genotypes (*p* = 0.013, Figure 4). The diminished expression of NF-κB1 in deletion allele carriers was also reflected by a 3-fold lower nuclear activity of the related subunit p50 48 h after LPS stimulation (*p* = 0.022, Figure 4). Strikingly, the mRNA expression and the nuclear activity of RelA (*p* = 0.517) and the related subunit p65 (*p* = 0.674) did not differ between II and ID/DD genotypes (Figure 4). All these results are mirrored in our findings in LPS-stimulated PBMCs of healthy subjects.

**Figure 5:**
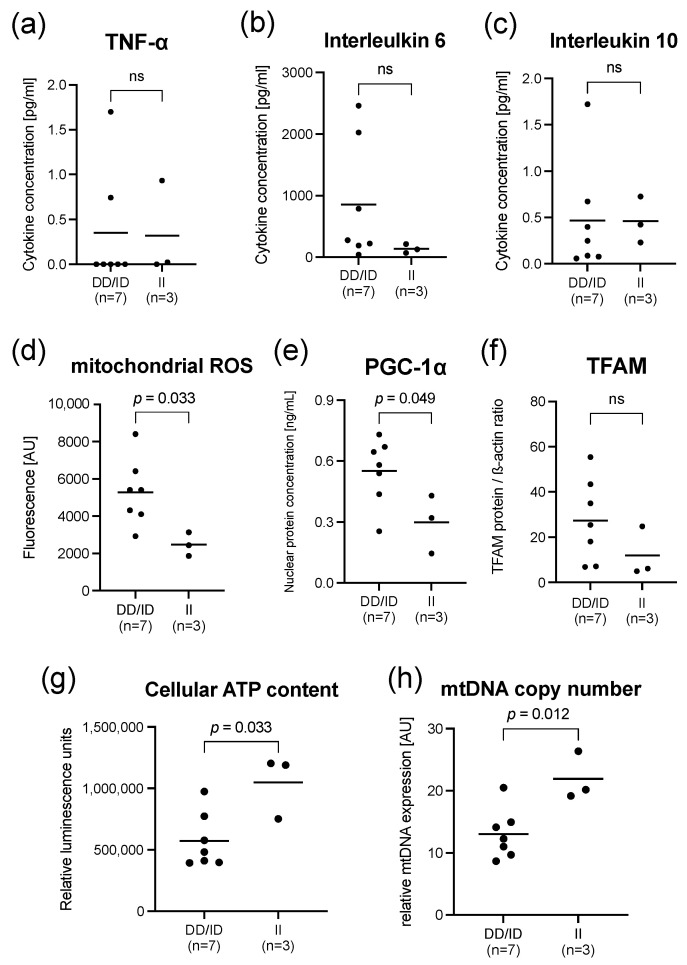
Sepsis is associated with an aggravated inflammation and intensified mitochondrial dysfunction in deletion allele carriers. Results representing PBMCs from septic patients (*n* = 10) sampled within 24 h after the diagnosis and stratified according to II-genotypes and ID/DD genotypes of the NF-κB1 promoter polymorphism. Upper panel: Concentrations of selected cytokines in PBMC cell culture supernatants. (**a**) TNF-α; (**b**) Interleukin 6; (**c**) Interleukin 10. Middle a lower Panel: (**d**) Mitochondrial ROS production in PBMCs from sepsis patients. (**e**) PGC-1α (ELISA of nuclear protein extracts) in PBMCs of healthy volunteers. (**f**) TFAM protein expression in PBMCs from sepsis patients. Mitochondrial function indicators (**g**) cellular ATP content and (**h**) mitochondrial DNA copy number in PBMCs from sepsis patients. Cellular ATP (**g**) was determined using a luciferase-based assay and expressed as relative fluorescent units normalized to 2.25 × 10^5^ cells per well. Each dot represents an individual patient; line represents mean. There were no missing data. *p*-values were determined using the Mann–Whitney test; ns designates no statistically significant difference. Cytokine concentrations pg/mL were derived from a calibration curve.

In line with the results from healthy volunteers, PBMCs of septic patients also tended to have higher concentrations of IL-6 in ID/DD genotypes compared to II genotypes (*p* = 0.183, Figure 5b). The TNF-α (*p* = 0.450) and IL-10 (*p* = 0.517) showed comparable serum concentrations between both genotype groups (Figure 5a,c). These findings do not contradict the results of our LPS endotoxin model (Figure 2), as the differences can be explained by a large heterogeneity in septic patients.

This was accompanied by an approximately 2-fold higher mitochondrial ROS production (*p* = 0.033) and an enhanced induction of mitochondrial biogenesis described by a higher nuclear PGC-1α protein concentration (*p* = 0.048) in the PBMCs, mimicking the results of our LPS endotoxemia model (Figure 5).

## 3. Discussion

This study highlights the deletion allele of the NF-κB1 (−94ins/delATTG) polymorphism being associated with an intensified inflammation and markedly aggravated mitochondrial dysfunction dependent on an altered composition of the NF-κB subunits. Our study unravels that a diminished expression of NF-κB1 and p50 DNA binding activity in D-allele carriers arises in both our LPS in vitro model and septic patients. This, in turn, suggests that a relative deficiency in p50 in sepsis paves the way to an intensified inflammation and more inflammation-associated collateral damages.

The NF-κB1 insertion–deletion (−94ins/delATTG) polymorphism has already been described as an important and independent prognostic factor for 30-day mortality, showing a remarkable hazard ratio of 2.3 regarding 30-day mortality in D allele carriers [5]. However, the molecular mechanisms have not been sufficiently understood, and the derivation of treatable traits according to this polymorphism was even contradictory. In this context, increased nuclear translocation of p65 was described as a cause in D-allele carriers without separately considering the nuclear translocation of the subunit p50 [5]. For this reason, an increased NF-κB gene expression in ID/DD genotypes was assumed to lead to an amplified activation of the NF-κB signaling pathway and, in turn, to the detrimental inflammatory response. The NF-κB-mediated inflammatory response can be inhibited by corticosteroids mainly via repression of p65-driven gene expression [17]; thus, corticosteroids should have been able to attenuate the inflammatory harm inflicted on D-allele carriers. However, in a subsequent study, hydrocortisone failed to abolish the inflammatory harm in deletion allele carriers and was even associated with increased 30-day mortality in septic shock [6]. Therefore, the overexpression of RelA or an increased nuclear translocation of p65 must be critically questioned as being the major cause of the intensified inflammation and mortality of ID/DD genotypes in sepsis.

Since the −94 ins/del ATTG polymorphism is near transcription factor binding motifs in the promoter region of the NF-κB1 gen, it is likely that the polymorphism primarily affects the expression of the encoding of the subunit p50. One of the first landmark studies by Karban and colleagues showed a decreased promoter activity in the NF-κB1 gene for the deletion allele that was associated with an increased risk of ulcerative colitis [18]. In addition, studies on tumors similarly demonstrated that the polymorphism in the promoter region of NF-κB1 primarily modulates the expression of NF-κB1 and its encoding subunit p50 without significantly affecting the expression of other subunits [19,20,21]. These findings are in line with our results showing for the first time that the ID/DD genotype of the NF-κB1 polymorphism in sepsis showed a diminished NF-κB1 expression and a reduced nuclear DNA binding activity of the subunit p50. Thus, we can confirm that, similar to septic patients, the altered nuclear activity of p50 is the main difference between II and ID/DD genotypes of the NF-κB1 polymorphism and probably the major cause for the subsequent inflammatory and metabolic derangements.

Interestingly, the baseline expression in healthy controls was not different between the genotypes, although our cohort with only 20 subjects is too small for a generalized assumption. Nevertheless, this finding offers a helpful explanation for the heterogenous observations in different studies regarding expression differences between genotypes and the clinical impact in association with the NF-κB1 polymorphism in different cohorts and diseases [22,23,24,25]. Therefore, it seems plausible that differences between the genotypes and the altered nuclear activity of p50 are mainly expressed under certain pathological conditions, such as sepsis, tumors, or diabetes mellitus [5,23,26]. This is supported by observations that different cytokine concentrations between the NF-κB1 genotypes were also mainly found under disease conditions. Different IL-6 concentrations between ID/DD genotypes, for example, were found in patients with myocardial infarction but not in control patients who had no history of cardiovascular diseases [27]. This is also in line with our observation by describing different TNF-α and IL-6 concentrations between the NF-κB1 genotypes after LPS stimulation and in septic patients, respectively, but not in unstimulated healthy controls. Taking these findings into account, we must assume that the NF-κB1 polymorphism leads to a relevant altered immune response with an amplified inflammatory insult in the deletion allele carriers only under certain conditions, such as sepsis.

Of course, the question arises why a diminished expression of the NF-κB1-subunit p50 leads to a higher inflammatory load in ID/DD genotypes. A potential explanation is included in the resulting decrease in p50 homo- and p50/p52 hetero-dimers. The subunits p50 and p52 lack a transcriptional transactivation domain, thus, are able to act as transcriptional repressors [28]. Therefore, they are competing with transcriptionally active dimers and, here, mainly p65 homo- and heterodimers. Consequently, the binding of p50 homo- and p50/p52 hetero-dimers to their DNA targets can mitigate the inflammatory response by competitive inhibition of ‘active’ p65 containing homo- and heterodimers. Support for this concept comes from animal experiments in which p50-deficient mice developed a more severe neutrophilic inflammation with a highly elevated TNF-α expression [29]. Furthermore, a recent study also reported an amplified degree of neuroinflammation in p50 knock-out mice together with an excessively increased TNF-α expression [30]. Therefore, a lack of nuclear p50 activity, as was present in D-allele carriers in our study, appears to be the main cause of the genotype-dependent aggravated inflammatory response in septic patients. Based on our findings, further investigations are warranted to clarify the exact mechanistic functions and interrelations between NF-κB1 subunits, especially p65 and p50, in sepsis. However, these important questions are beyond the scope of this study and need to be clarified by upcoming studies.

Previous investigations showed that the deletion allele of the NF-κB1 polymorphism was associated with increased disease severity in sepsis and acute respiratory distress syndrome [5,16]. However, our results cannot reliably confirm that the D allele is indeed associated with aggravated disease severity of sepsis, presumably due to our small sample size. Nevertheless, our study unravels some important molecular mechanisms by which the D allele of the NF-κB1 polymorphism seems to orchestrate the amplified inflammatory response in aggravated organ dysfunction in sepsis. A dysregulated NF-κB composition and nuclear activity can contribute to the pathogenic processes of sepsis and lead to devastating perturbations for the host [8]. Here, oxidative stress, and especially mitochondrial ROS production, is one of the major elements that is affected by altered inflammation and significantly contributes to organ dysfunction in septic patients [31,32]. It must be noted that ROS, in general, is essential for many life-sustaining processes in cells and tissues, and its liberation is basically a physiological response [33]. However, they can also show detrimental effects, especially when the activities are uncontrolled and exceed a certain threshold, potentially leading to significant mitochondrial injury [32,34].

Importantly, we could demonstrate that the NF-κB1 polymorphism had a dramatic impact on the mitochondrial ROS production, with a more than 2-fold higher mitochondrial ROS production in ID/DD genotypes. In this regard, we can consistently show that the exaggerated mitochondrial ROS production in the D-allele carriers was accompanied by an aggravated mitochondrial dysfunction, described by a decrease in the cellular ATP content and a diminished mtDNA copy number. Therefore, our results elucidated new insights into mechanisms explaining why the NF-κB1 insertion–deletion (−94ins/delATTG) polymorphism significantly impacts disease severity, outcome, and mortality in sepsis.

Finally, the question also arises as to why cortisone in deletion allele carriers has a detrimental effect on the outcome of sepsis [6]. Although the mechanistic interrelations need to be revealed by subsequent studies, we can make some preliminary considerations based on our findings. Although glucocorticoid actions are mainly described by anti-inflammatory properties, studies showed that glucocorticoids can also exert pro-inflammatory effects, and a glucocorticoid treatment could exacerbate the inflammatory immune response [35]. We can speculate here that these pro-inflammatory effects of glucocorticoids may be more intensively exerted in deletion allele carriers. Another interesting aspect comes from experiments with NF-κB1 knock-out mice. Mice lacking the p50 subunit showed profound defects in immune cell function [36]. Moreover, from this point of view, additional treatment with glucocorticoids may further aggravate immune cell dysfunction, which could explain the higher mortality observed in ID/DD genotypes dependent on cortisone treatment.

## 4. Materials and Methods

### 4.1. Study Design and Oversight

We conducted a prospective, observational, single-center, in vitro, and in vivo study. This study was reviewed and approved by the Ethics Committee of the Medical Faculty of the Ruhr-University of Bochum (protocol no. #18-6257), and written informed consent was obtained from healthy subjects, patients, or their guardians, as appropriate. This study was conducted in accordance with the Declaration of Helsinki, good clinical practice guidelines, and local regulatory requirements.

### 4.2. Patient and Volunteer Cohorts and Treatments

We recruited twenty healthy subjects between October 2018 and December 2019 who were free from infection for at least 4 weeks prior to study participation. Blood was drawn and PBMCs isolated as described below. For subsequent experiments, PBMCs of healthy subjects were seeded at a density of 2 × 10^7^ cells per well and incubated with a concentration of 10 µg/mL LPS (*Escherichia coli* type 0111:B4; L4391, Sigma-Aldrich, St. Louis, MI, USA). In contrast, PBMCs of septic patients were not treated with LPS. Measurements were performed without LPS stimulation (control) and at 0.5, 4, 24, and 48 h after LPS stimulation.

Septic patients were considered eligible if they fulfilled the criteria of the Sepsis-3 definition [1]. Furthermore, enrollment, written informed consent, and blood sampling had been completed within the first 24 h after diagnosis of sepsis. Exclusion criteria were age under 18 years, pregnancy, pre-existing anemia, known mitochondrial disorder, and the decision to withhold or withdraw life-sustaining treatment on the day of study inclusion. Ten septic patients admitted to the intensive care unit (ICU) of the University Hospital Knappschaftskrankenhaus Bochum between December 2018 and February 2019 were included. Blood samples of septic patients were also utilized in our previously published study [37]. PBMCs of septic patients were isolated as described below. Cells were not stimulated and directly processed for subsequent PBMC isolation.

### 4.3. Isolation of Peripheral Blood Mononuclear Cells

Peripheral blood mononuclear cells (PBMCs) were isolated using a density gradient centrifugation protocol (Ficoll Paque solution, GE Healthcare Bio Science AB, Uppsala, Sweden) as described previously [37]. Briefly, cells were centrifuged in Ficoll Paque solution, and the PBMC-rich layer was collected. PBMCs of septic patients were directly processed for further experiments. We resuspended isolated cells of healthy volunteers in full RPMI 1640 medium (Invitrogen, Waltham, MA, USA) containing 10% fetal calf serum (Biochrom AG, Berlin, Germany), 100 U/mL penicillin plus 100 μg/mL streptomycin (both Invitrogen, USA). Cultured and LPS stimulated cells were held at 37 °C in a humidified atmosphere containing 5% CO_2_.

### 4.4. NF-κB1 Genotyping

The genomic DNA of patients was extracted from whole blood using standard methods (QIAamp, QIAGEN, Hilden, Germany). The −94ins/delATTG NF-κB1 insertion–deletion (−94ins/delATTG) promoter polymorphism (rs28362491) was determined restriction analysis. Therefore, the primer NF-κB1 insertion–deletion (−94ins/delATTG) polymorphism _del/ins_SE (5′-CTTGGATCCATGCCGACCC-3′) and NF-κB1 insertion–deletion (−94ins/delATTG) polymorphism _del/ins_AS (5′-TAGGGAAGCCCCCAG-GAAG-3′) were used to amplify a 158 bp PCR fragment, followed by digestion with restriction enzyme PFiMI (New England Biolabs, Ipswich, PA, USA). The PCR was performed at an annealing temperature of 60 °C in a 25 µL commercially available PCR master mix (New England Biolabs). The analysis was performed using agarose gel electrophoresis (PeqLab, Erlangen, Germany), resulting in a 158 bp fragment for D-allele and a 121 bp product for I-allele. Randomly chosen samples with known genotype were reanalyzed for result verification.

### 4.5. mRNA Expression of NF-κB1, RelA, and Determination of mtDNA Copy Number

Total DNA and RNA were extracted from PBMCs using the QIAamp and RNeasy kits, respectively, according to the manufacturers’ instructions (QIAGEN, Germany), to assess the gene products by quantitative polymerase chain reaction (PCR). The purified RNA in mRNA samples was reverse transcribed into complementary DNA using the QuantiTect Reverse Transcription Kit (QIAGEN, Hilden, Germany). The PCR was performed in duplicate using the GoTaq1 PCR Master Mix (Promega, Madison, WI, USA) and the subsequent primers (Table 2) on a CFX Connect Real-Time System (Bio-Rad Labs, Hercules, CA, USA).

Relative mRNA expression was calculated after normalization using beta actin as internal controls utilizing the 2^−ΔCT^ method. The mitochondrial DNA copy number was quantified as the ratio of DNA products of mitochondrial nicotinamide adenine dinucleotide dehydrogenase subunit 1 normalized to ribosomal 18S-RNA serving as an internal control using the 2^−ΔCT^ method, as described previously [37].

### 4.6. DNA-Binding ELISA Measuring NF-κB Activity

The p50, p52, p65, c-Rel, and RelB DNA binding activity were measured by TransAM transcription factor assay kits (Active Motif, Carlsbad, CA, USA). Nuclear extracts were prepared in lysis buffer AM2 (Active Motif, USA). Nuclear extracts were incubated with the immobilized consensus sequence, and each subunit was detected using specific antibodies, according to the manufacturers’ instructions.

### 4.7. Cytokine Concentrations

The supernatant of PBMCs was collected and used for quantifying the cytokines TNF-α, interleukin 6 (IL-6), and interleukin 10 (IL-10) utilizing ELISA kits (BioLegend, San Diego, CA, USA), according to the manufacturer’s instructions. Each cytokine concentration was derived by applying respective calibration standard curves.

### 4.8. Nuclear Concentration of PGC-1α

In order to quantify the nuclear concentration of peroxisome proliferator-activated receptor-gamma coactivator 1-alpha (PGC-1α), cells were centrifuged at 4000× *g*, and the resultant pellet was then resuspended in Pre-Extraction Buffer (Abcam, Cambridge, UK), allowing the cells to swell on ice. After vortexing and further centrifugation, the pellet was dissolved in Complete Lysis Buffer (Active Motif, USA). The nuclear concentrations of PGC-1α were measured using a dedicated human ELISA kit (Wuhan EIAab Science Co., Wuhan, China), according to the manufacturer’s instructions.

### 4.9. Mitochondrial Reactive Oxygen Species Production

Mitochondrial ROS in PBMCs was determined using the fluorescent MitoSOX™ MitoSOX™ Red Mitochondrial Superoxide Indicator (Thermo Fisher, Bremen, Germany). Then, 5 μM MitoSOX was incubated with 2.5 × 10^6^ PBMC diluted in Dulbecco’s phosphate-buffered saline for 10 min at 37 °C in darkness. Afterward, cells were washed and centrifuged (1600× *g* rpm, 10 min). Excitation was set at 510 nm and emission at 580 nm. A positive control group of mitochondrial ROS production was elaborated with 1-methyl-4-phenylpyridinium.

### 4.10. Cellular ATP Content

We performed a luciferase-based assay (Cell Titer Glo 2.0 Assay, Promega, USA), following the manufacturer’s instructions, to assess the cellular ATP content. Accordingly, 90 μL of the cell suspension (2.5 × 10^6^ PBMC/mL) was used per well. After the degree of cytotoxicity was assessed using the CellTox Green cytotoxicity assay (Promega, USA), the ATP content was measured using the CellTiter Glow 2.0 reagent (Promega, USA). The luminescence was recorded subsequently.

### 4.11. Cellular TFAM Protein Concentration

Western blot was used to assess TFAM protein concentrations as described previously [37]. The membranes were probed with the primary antibodies against TFAM (1:200; sc-376672, Santa Cruz Biotechnology, Dallas, TX, USA) and beta actin (1:10,000; Millipore, Burlington, MA, USA) for five hours at room temperature. Subsequently, incubation of secondary antibodies (1:10,000; Goat-anti-mouse, IRDye680RD, and 1:10,000; donkey-anti-rabbit, IRDye 800cw, both Li-cor Biosciences, Lincoln, NE, USA) for one hour at room temperature followed. Protein bands were visualized with an Odyssey Scanner (Li-cor Biosciences). The densitometry was determined using Image J 2.0 software (National Institutes of Health, Bethesda, MD, USA).

### 4.12. Statistical Analysis

The characteristics of the patients are reported as percentages for categorical variables and as means with SD or medians with interquartile ranges (25th and 75th percentile), as appropriate. Categorical variables were compared using McNemar or Fisher’s exact tests. Continuous independent variables were compared using the Student’s *t*-test or the Mann–Whitney U test. Continuous dependent variables were compared using the paired samples Student’s *t*-test or the Wilcoxon signed-rank test, as appropriate. Differences between genotypes in time series measurement were each conducted as mixed-effects analysis with post hoc Šídák’s multiple comparisons test. Heterozygous and homozygous deletion allele carriers were combined in our analysis of healthy volunteers and in septic patients due to the low frequency of DD genotypes. A *p*-value of less than 0.05 was considered statistically significant. All analyses were performed using SPSS (version 25, IBM, Chicago, IL, USA). GraphPad Prism 9 (Graph-Pad, San Diego, CA, USA) was used for graphical presentations.

## 5. Conclusions

Our results enlighten the important mechanistic role of the NF-κB1 promoter polymorphism in sepsis. We showed here that the deletion allele of the NF-κB1 promoter polymorphism in sepsis reduces the expression and nuclear activity of subunit p50, which was, in turn, associated with an amplified inflammatory response, increased ROS production, and an aggravated mitochondrial dysfunction. In conclusion, our results underpin the important role of genotype-dependent patterns as essential traits in sepsis.

### Limitations

Limitations of this investigation should also be mentioned. Unrecognized selection bias, inherent to many genetic association studies, cannot ultimately be excluded. Moreover, although all sepsis patients were treated with a rather standardized multimodal regimen, we cannot exclude that unknown confounding factors exist due to the multifactorial nature of this disorder. Furthermore, the study population was rather small; thus, reliable data regarding disease severity impact on outcome and mortality cannot be derived from our data. In addition, the inherent biological variability in our septic patients concerning our small sample size must also be considered critically regarding our mechanistic conclusions. However, as we were able to mimic the in vivo observations in septic patients with our LPS endotoxin model, we are confident that this underpins the potential relevance of our findings.

## Figures and Tables

**Table 1:** Baseline characteristics of sepsis patients stratified by their NF-κB1 insertion–deletion (−94ins/delATTG) polymorphism.

Variable	ID/DD Genotypes (*n* = 7)	II Genotypes (*n* = 3)	*p* Value
Age [yrs.], mean (±SD)	57 ± 15	60 ± 10	0.909
Male sex, *n* (%)	4 (57%)	2 (66%)	0.778
Body mass index (kg/m^2^)	28.3 ± 3.3	24.3 ± 4.0	0.137
Site of infection, *n* (%)			0.774
-Pneumonia	3 (43%)	2 (67%)	
-Abdominal infection	2 (29%)	1 (33%)	
-Skin and soft tissue infection	1 (14%)	-	
-Urinary tract infection	1 (14%)	-	
Culture results, *n* (%)			0.116
-Gram-positive isolates only	3 (43%)		
-Gram-negative isolates only	3 (43%)	1 (33%)	
-Mixed bacterial isolates	-	1 (33%)	
-Negative cultures	1 (14%)	1 (33%)	
C-reactive protein concentration (mg/dL), mean (±SD)	25.8 ± 8.9	16 ±16.1	0.305
Procalcitonin concentration (ng/mL), mean (±SD)	14.7 ± 28.8	6.0 ± 7.9	0.627
Leukocyte concentration (10^9^/L), mean (±SD)	16.7 ± 5.9	18.6 ± 13.2	0.909
Simplified Acute Physiology Score, mean (±SD)	48 ± 19	42 ± 21	0.668
SOFA score, mean (±SD)	11 ± 4	9 ± 6	0.545
Renal replacement therapy, *n* (%)	4 (57%)	1 (33%)	0.490
Mechanical ventilation, *n* (%)	3 (43%)	1 (33%)	0.778
Serum lactate concentration (mg/dL), mean (±SD)	1.8 ± 1.0	1.6 ± 1.1	0.817
Vasopressor support, *n* (%)	7 (100%)	2 (66%)	0.646
Death within 30 days, *n* (%)	3 (43%)	1 (33%)	0.778

Data are presented as *n* (%) or mean (±SD), as appropriate. The presented characteristics refer to baseline measurements recorded on study inclusion. There were no missing data. SOFA score—Sepsis-related Organ Failure Assessment score.

**Table 2:** Oligonucleotide pairs used for quantitative polymerase chain reaction.

Oligonucleotide Name	Sequence
mRNA (c-DNA) targets	
RelA (p65) forward	5′-GCGAGAGGAGCACAGATACC-3′
RelA (p65) reverse	5′-GGGGTTGTTGTTGGTCTGGA-3′
NF-κB1 (p50) forward	5′-GTGAAGGCCCATCCCATGGT-3′
NF-κB1 (p50) reverse	5′-TGTGACCAACTGAACAATAACC-3′
Beta actin forward	5′-CATGTACGTTGCTATCCAGGC-3′
Beta actin reverse	5′-CTCCTTAATGTCACGCACGAT-3′
DNA targets	
Mitochondrial NADH dehydrogenase subunit 1 forward	5′-CACCCAAGAACAGGGTTTGT-3′
Mitochondrial NADH dehydrogenase subunit 1 reverse	5′-TGGCCATGGGTATGTTGTTAA-3′
18SrRNA forward	5′-TAGAGGGACAAGTGGCGTTC-3′
18SrRNA reverse	5′-CGCTGAGCCAGTCAGTGT-3′

## Data Availability

The data presented in this study are available on request from the corresponding author. The data are not publicly available due to local legal restrictions.
